# PReS-FINAL-2215: Genotype-phenotype correlations in children with Familial Mediterranean Fever in Germany

**DOI:** 10.1186/1546-0096-11-S2-P205

**Published:** 2013-12-05

**Authors:** F Gohar, M Jeske, P Lohse, T Kallinich, U Neudorf, T Niehues, H Wittkowski, E Lainka, D Foell

**Affiliations:** 1 Department of Paediatric Rheumatology and Immunology, University of Muenster, Muenster, Germany; 2 Paediatric Rheumatology, Children's Clinic, Uniklinikum Essen, Essen, Germany; 3 Labor Blessing und Partner, Molekulargenetik, Singen, Germany; 4 Department of Pediatric Pneumology and Immunology, Department of Rheumatology, Charité - Universitätsmedizin Berlin, Berlin, Germany; 5 Paediatric Rheumatology, University of Duisburg-Essen, Essen, Germany; 6 Center for Child and Adolescent Medicine, HELIOS Hospital Krefeld, Krefeld, Germany; 7 Paediatric Rheumatology, University Hospital Essen, Children's Hospital, Essen, Germany

## Introduction

Familial Mediterranean fever (FMF) is one of the most common autoinflammatory diseases (AID). A variety of relevant mutations in the MEFV gene have been demonstrated. Pro-inflammatory S100 proteins correlate with disease activity in autoinflammatory disorders, and have been previously correlated with clinically active FMF. Here, we describe the association between these biomarkers and stable FMF including different mutations, as recorded in the German AID-Net-registry.

## Objectives

Our objectives were to 1) analyse genotype-phenotype correlations and 2) describe mutation-specific associations with associations with MRP8/14 and S100A12 biomarker results, in stable FMF patients.

## Methods

We used two common scoring systems modified for children (Mor et al., Pras et al.) to assess disease severity in 243 FMF patients of the AID-Net-registry. For the five most frequent mutations, we tested for a correlation of the genotype with the phenotype, mean CRP, and ethnic origin, respectively. Patients were sub-grouped by mutation and their MRP8/14 and S100A12 biomarker results were evaluated. Statistical significance was tested using SPSS.

## Results

Among the 243 patients, we detected a total of 433 pyrin mutations and 22 different sequence variants, including one new mutation (p.Gly488Asp). The five most frequent alterations were p.Met694Val (55%, n = 238), p.Met680lle (12%, n = 52), p.Val726Ala (10%, n = 44), p.Glu148Gln (8%, n = 34) and p.Met694Ile (2,3%, n = 10). P.Met694Val in homozygous form (30%, n = 73) was correlated with a more severe disease activity, based on the score by Mor, as well as with a higher mean CRP (74 versus 31 mg/l) compared to patients without this mutation (p = 0.01 and p < 0.01, respectively). The score suggested by Pras did not yield a significant genotype-phenotype correlation; indeed, the two scoring systems were inconsistent with each other (r < 0.07). Patients with any M694V gene mutation did not have different MRP8/14 concentrations than patients without M694V mutations (mean 5,830 versus 2,640 ng/ml; p = 0.88). Patients with any M694V gene mutation did also not have different S100A12 levels (1,880 vs 495 ng/ml; p = 0.39). M694V heterozgotes also did not differ from M694V homozygotes in either MRP8/14 or S100A12 levels (p = 0.81 and p = 0.74, respectively).

## Conclusion

The homozygous p.Met694Val substitution was associated with a more severe disease activity. We did not find any statistically significant differences in the MRP8/14 or S100A12 levels between patients with and without M694V mutations, or between M694V heterozygotes or homozygotes in those with stable FMF. The well-known severity scores for children (Mor, Pras) are inconsistent. The AID-Net is working on a new scoring system.

## Disclosure of interest

None declared.

